# Evaluation of fluorimetric assay conditions for measuring leucine aminopeptidase activity in soils

**DOI:** 10.1371/journal.pone.0352890

**Published:** 2026-07-07

**Authors:** Qingqing He, Shunyu Huang, Jipeng Wang

**Affiliations:** 1 School of Emergency Management, Xihua University, Chengdu, China; 2 Mountain Ecological Restoration and Biodiversity Conservation Key Laboratory of Sichuan Province, Chengdu Institute of Biology, Chinese Academy of Sciences, Chengdu, China; 3 State Key Laboratory of Hydraulics and Mountain River Engineering, College of Water Resources and Hydropower, Sichuan University, Chengdu, China; 4 Sichuan Geological Environment Survey and Research Center, Chengdu, China; São Paulo State University Institute of Biosciences Languages and Exact Sciences: Universidade Estadual Paulista Julio de Mesquita Filho Instituto de Biociencias Letras e Ciencias Exatas, BRAZIL

## Abstract

Leucine aminopeptidase (LAP) plays a crucial role in the hydrolysis of proteinaceous nitrogen in soils. However, existing studies use varying conditions in the fluorimetric assay of soil LAP, which hinders cross-study comparison of the measured activities. Using one purified enzyme and three soils of contrasting properties, we examined how LAP activity responded to variations in assay conditions including buffer pH, substrate (L-leucine-7-amido-4-methylcoumarin) concentration, temperature, incubation time, soil amount, and metal ion concentrations. We found that: (1) the optimal pH for LAP activity ranged from 7 to 9; (2) a substrate concentration of 250 μM was necessary to achieve zero-order reaction kinetics; (3) LAP activity increased as the temperature rose from 10 to 40 °C, with a Q_10_ value between 1.63 and 2.45; (4) the rate of the enzymatic reaction remained stable for at least 3 hours; (5) measured activity decreased as the amount of soil used for homogenate preparation increased from 0.5 to 2.0 g; and (6) the activity of LAP was not substantially stimulated by the addition of metal ions, suggesting that a metal cofactor is not needed for the fluorimetric assay of LAP. We also compared the behaviors of LAP with those of the colorimetrically measured arylamidase that catalyze the release of an N-terminal amino acid from peptides, amides, or arylamides, and found that they may represent the same group of soil enzymes. Our findings may help standardize the assay protocol for soil LAP, which is essential for conducting meta-analysis of enzyme activities measured across different studies.

## 1. Introduction

Soil leucine aminopeptidase (LAP, Enzyme Commission Number: 3.4.11.1) catalyzes the hydrolysis of amino acid residues from the N-terminus of peptides [1]. The fluorogenic substrate L-leucine-7-amido-4-methylcoumarin (leucine-AMC) is commonly used to assess soil LAP activity, allowing researchers to investigate the potential rate of proteinaceous nitrogen (N) hydrolysis in soils [2,3]. Furthermore, many studies have used LAP activity, often in conjunction with that of β-1,4-N-acetylglucosaminidase, as an indicator of microbial investment in N acquisition [1,4–6]. Specifically, LAP assays are particularly important for soil ecological stoichiometry studies, where LAP activity is often compared with C- and P-acquiring enzymes to infer microbial nutrient limitation across ecosystems [7]. Given that both plants and soil microbes largely rely on hydrolyzed amino acids or subsequently mineralized inorganic N for their growth, establishing a standardized assay procedure for soil LAP activity is crucial for understanding the regulators of soil N availability.

The fluorimetric assay conditions for soil LAP, including pH, temperature, substrate concentration, incubation time, and soil amount, vary considerably among widely cited studies [8–12]. For instance, the buffer pH ranges from acidic to alkaline (5.0 to 7.8), and substrate concentrations can differ by nearly an order of magnitude (50–400 μM) [10,12]. The absence of a standardized assay protocol complicates cross-study comparisons and meta-analysis of the measured activities [11]. Additionally, measuring enzyme activities under suboptimal conditions may reduce assay sensitivity, increasing the risk of type II errors when detecting treatment effects on enzyme activity [9,11]. Therefore, a comprehensive understanding of how various factors influence the fluorimetric assay for LAP is essential for developing a standardized protocol.

Previous studies have established a colorimetric assay protocol for enzymes that catalyze the hydrolysis of soil organic N [13]. This method uses the substrate L-leucine β-naphthylamide (LNA) and designates the measured enzyme as “arylamidase” (EC: 3.4.11.2). Because leucine-AMC is similar to LNA in that the leucine moiety is linked to an aromatic ring in an arylamide structure, it is plausible that the soil LAP measured in the fluorimetric assay represents the same group of enzymes as the arylamidase. If this is the case, enzyme activities measured with these two substrates in different publications could be compared and compiled. The detailed characterizations of soil arylamidase in previous studies [13–15] provide an opportunity to compare LAP and arylamidase in their behaviors.

In this study, we investigated the factors that may influence the fluorimetric assay of soil LAP by using three soils collected from different regions of China with contrasting properties and a purified LAP from porcine kidney. The purified enzyme is assayed without interference from soil particles, allowing it to serve as a reference for soil enzymes. Additionally, we compared the LAP measured using leucine-AMC with the arylamidase activity measured using LNA to determine whether they represent the same group of soil enzymes.

## 2. Materials and methods

### 2.1. Purified enzyme and soils

The purified LAP enzyme was purchased from Sigma (product number: L5006, microsomal from porcine kidney, 31 mg enzyme mL^-1^ in an ammonium sulfate suspension, stored at 4 °C). Three soils with contrasting properties were used in the present study (Table 1). Soils 1 and 2 were collected from the Yingtan Station (28°15′ N, 116°55′ E) and Yanting Station (31°16′ N, 105°27′ E), respectively, of the Chinese Ecosystem Research Network (CERN). Soil 3 was collected from the Mulun National Nature Reserve (107°59′ E, 25°08′N). We acquired soil sampling permits respectively from Yanting Purple Soil Agricultural Ecology Experimental Station (Chinese Academy of Sciences, CAS), Yingtan Red Soil Ecological Experimental Station (CAS), and Hechi Municipal Bureau of Forestry. At each site, approximately 1 kg of soil samples were collected from the top 10-cm layer and passed through a 2-mm sieve. The fresh soil samples were stored at 4 °C until enzyme assay within one month. Air-dried soils were used for pH analysis with a soil:water ratio of 1:2.5. For particle size distribution analysis, air-dried soil samples were pretreated with 30% H_2_O_2_ and 10% HCl to remove organic matter and carbonates, respectively. The soil aggregates were then dispersed by adding 0.05 M sodium hexametaphosphate and stirred, and the particle size distribution was analyzed by a Malvern MS 2000 using the laser diffraction method (Malvern Instruments, Malvern, England). Oven-dried (60 °C) soils were ground and pretreated with 0.5 M HCl to remove carbonate, and then analyzed for total organic C and total N using an element analyzer (Elementar vario MACRO cube, Germany).

**Table 1:** Information of the soils used for evaluating the fluorimetric assay conditions of leucine aminopeptidase.

	Soil 1	Soil 2	Soil 3
pH	5.55	7.63	7.19
SOC (mg g^-1^)	10.2	43.1	59.9
TN (mg g^-1^)	1.27	2.68	3.46
Clay/silt/sand (%)	40.78/34.38/24.83	3.20/63.68/33.13	18.79/70.58/10.63
Parent material	Quaternary red clay	Purple sandstone/mudstone	Limestone and dolomite
WRB classification	Ferralsols	Cambisols	Regosols
Vegetation	Cropland	Secondary forest	Secondary forest
MAT	17.6 °C	17.5 °C	19.5 °C
MAP	1795 mm	826 mm	1422 mm

Abbreviations: SOC, soil organic carbon; TN, total nitrogen; WRB, world reference base; MAT, mean annual temperature; MAP, mean annual precipitation.

### 2.2. Enzyme assay

For fluorimetric assays, three replicate homogenates were used for each soil and four replicates were used for the purified enzyme. Soil homogenate was prepared by placing fresh soils into a 150-mL beaker on a stir plate, adding 120 mL deionized water and then stirring at 600 rpm for 30 min with a 3.5-cm magnetic stir bar. During stirring, the soil homogenate was taken up with a multiple-channel pipette set at 100 μL from the middle of the beaker, and placed into microplate wells containing 50-μL tris(hydroxymethyl)methyl aminomethane (THAM) buffer (100 mM, pH 8). The top of the pipette tip was cut off to reach an inner diameter of ~3 mm so that all the soil particles could be taken up. Subsequently, 50 μL of substrate solution (1 mM leucine-AMC) was added to each well, which brought the final reaction volume to 200 μL. The mixtures were incubated at 37 °C in the dark without shaking for 1 h before immediate (within 1 min) fluorescence reading by a fluorometer (Thermo Varioskan™ LUX, Finland) with 365 nm excitation and 450 nm emission filters. A reference standard [50-μL buffer, 100-μL water, and 50-μL 10-μM 7-amido-4-methylcoumarin (AMC)], quench standard (50-μL buffer, 100-μL soil homogenate, and 50-μL 10-μM AMC), soil control (50-μL buffer, 100-μL soil homogenate, and 50-μL water), and substrate control (50-μL buffer, 100-μL water, and 50-μL 1-mM leucine-AMC) were prepared for each sample. There were six replicates for the assay, reference, and control wells. The enzyme activity was calculated according to German et al. [9]. The substrate solution and the microplate containing soil homogenates were preincubated at the assay temperature for 20 min before mixing, in order to maintain the reaction mixture at the assay temperature throughout the incubation procedure [11].

The assay of purified enzyme was the same as that applied to the soil homogenate, except that the 100-μL soil homogenate was replaced with 100-μL diluted enzyme suspension. While purified porcine LAP provides a useful reference standard, soil LAP is produced by diverse microbial communities and may therefore exhibit distinct kinetic properties. Accordingly, careful validation of assay conditions in soil matrices remains essential, as is the case for other fluorogenic detection systems [9]. To prepare the diluted enzyme solution, the purchased enzyme suspension was first brought to 5 mL with 3.5-M ammonium sulfate solution, and the obtained enzyme suspension (stock suspension) was stored at 4 °C. To activate the enzyme, 0.1-mL stock suspension was mixed with 0.9-mL THAM buffer (100 mM, pH 8) and incubated at 37 °C for 2 h according to Bisswanger [16] with slight modifications. In Bisswanger [16], the buffer solution used was THAM buffer (pH 8.5) containing 1.25 mM MnCl_2_. Here, we changed it to THAM buffer (pH 8) without MnCl_2_ in order to be consistent with the assay condition of soil homogenate. The activated enzyme suspension was diluted 5000 times for activity assay. Preliminary experiments showed that the diluted purified enzyme and the soils used in this study catalyzed the hydrolysis of leucine-AMC at comparable rates. The enzyme suspension was sufficiently mixed before each dilution and addition. The activated enzyme suspension was used within 12 h.

The effect of the assay pH on LAP activity was examined in the pH range of 5–10. Since THAM is unable to effectively buffer solution pH beyond the range of 7–9, a modified universal buffer (50 μL, 100 mM; [17]), which is capable of buffering reaction pH values in the tested range, was used in place of the THAM buffer. This allows the effect of pH to be assessed in the same chemical solution, which is important because ionic composition of the buffer may influence enzyme activity [17,18]. It should be noted that the modified universal buffer was used only for the evaluation of pH optima, and THAM was used for the other experiments (assayed at pH 8).

The effect of substrate concentration was tested using eight concentrations of leucine-AMC ranging from 0 to 500 μM. In addition, seven concentrations of L-alanine-7-amido-4-methylcoumarin (alanine-AMC) and of L-serine-7-amido-4-methylcoumarin (serine-AMC) ranging from 0 to 375 μM were used to compare LAP and arylamidase in terms of substrate specificity [15]. The concentration ranges of the three substrates were chosen based on our preliminary experiments to ensure that V_max_ can be achieved.

The effect of temperature was tested using seven temperatures ranging from 10 to 40 °C according to Razavi et al. [19]. We did not conduct LAP assay at high temperatures because the fluorescence of AMC decreased at temperatures higher than 40 °C possibly due to increased frequency of molecular collision and energy transfer at high temperatures (S1 Fig; [20]).

The effect of incubation time was tested by monitoring the amount of AMC released for 3 h, which encompasses the commonly adopted incubation time of 1 h in enzyme assays [21,22]. Based on the soil amount adopted in previous studies for homogenate preparation [23], the effect of soil amount was tested in the range of 0.5 to 2 g soil per 120 mL.

The effects of Co^2+^ (CoCl_2_·6H_2_O), Mn^2+^ (MnCl_2_), Mg^2+^ (MgCl_2_·6H_2_O), Cd^2+^ (CdCl_2_), Ag^+^ (AgNO_3_) and B_4_O_7_^2-^ (Na_2_B_4_O_7_) were examined to determine if co-factors are required in the LAP assay and to compare LAP and arylamidase in terms of their responses to different ions [14]. Fifty-μL solutions with eight ion concentrations ranging from 0 to 5000 μM (corresponding to final concentration of 0–1000 μM) were added to the microplate containing soil homogenates. After equilibration for 20 min at room temperature, the soil-ion mixtures were incubated with the substrate solution for 1 h at 37 °C.

To compare LAP and arylamidase in terms of pH optimum, reaction kinetics and temperature dependence, we followed Acosta-Martínez and Tabatabai [13] to measure the arylamidase activity using the chromogenic substrate LNA. The only modification was that the incubation was conducted without shaking to be comparable to the fluorimetric assay. The effects of assay pH, LNA concentration, and temperature on arylamidase activity were examined using the purified enzyme and the three soils. Three replicates were performed for each colorimetric assay.

### 2.3 Statistical analysis

Differences between treatments were analyzed with ANOVA followed by a Bonferroni post hoc test (SPSS statistical software, version 22.0, IBM, USA). Values of *P* < 0.05 were considered to be significant. The dependences of the measured enzyme activity on substrate concentration and temperature were fitted with the Michaelis-Menten equation (Equation 1) and the logarithmic form of the Arrhenius equation (Equation 2), respectively, and curve fittings were performed using the Origin 2016 software (OriginLab, USA).


$$\begin{array}{c} Activity=V_{max}[S]/(K_{m}+[S]) \end{array}$$ (1)

where Activity is the measured enzyme activity, V_max_ is the maximum enzyme activity, K_m_ is the half saturation constant, the substrate concentration at which the enzyme activity equals V_max_/2, [S] is the substrate concentration.


$$\begin{array}{c} \text{ln}(Activity)=-\frac{E_{a}}{RT}+\text{ln}(A) \end{array}$$ (2)

where Activity is the measured enzyme activity, A is the pre-exponential factor, E_a_ is the activation energy, R is the gas constant, and T is the absolute temperature in K.

## 3. Results

### 3.1. pH dependence

**Fig 1:**
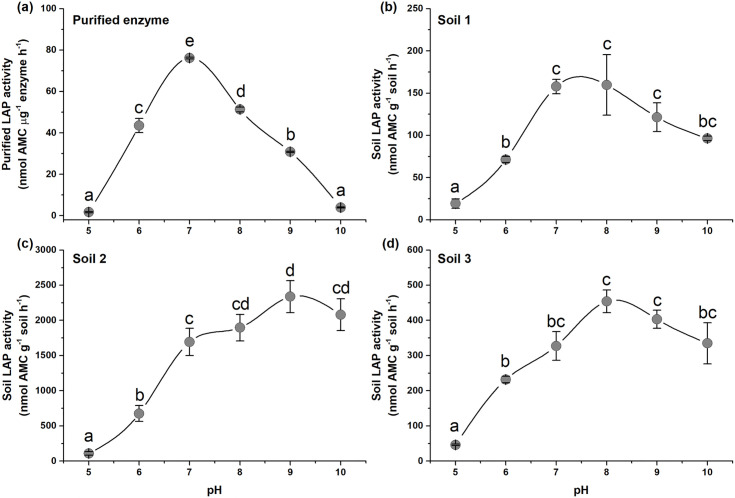
Effect of buffer pH on LAP activity. Data are presented as mean ± standard error. Different lowercase letters indicate significant differences among different buffer pH values. Note that the unit and the number range in the y-axis were not the same for the four panels. LAP: leucine aminopeptidase, AMC: 7-amido-4-methylcoumarin.

In the fluorimetric assays, the activity of purified LAP was the highest at neutral pH and decreased markedly toward the acidic and alkaline ends of the tested pH values (Fig 1a). The activities of soil LAP were the highest at pH values of 7–9 and were significantly higher under alkaline conditions than under acidic conditions (Fig 1b–1d). For the same purified LAP and soils, the enzyme activities measured colorimetrically using LNA also showed pH optima of 7–9 (S2 Fig).

### 3.2. Kinetic parameters and substrate specificity

**Fig 2:**
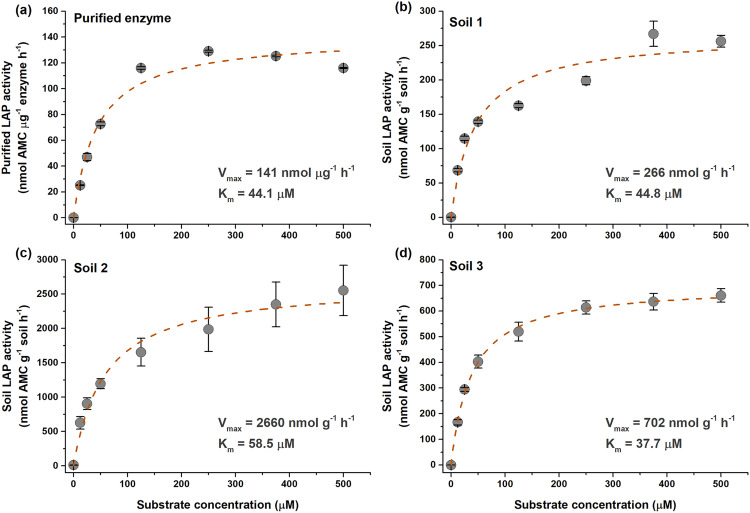
Effect of substrate concentration on LAP activity. Data are presented as mean ± standard error. The relationship between substrate concentration and enzyme activity was fitted by the Michaelis-Menten equation. Note that the unit and number range in the y-axis were not the same for the four panels. V_max_: maximum enzyme activity, K_m_: half saturation constant, the substrate concentration at which the enzyme activity equals V_max_/2, LAP: leucine aminopeptidase, AMC: 7-amido-4-methylcoumarin.

In the fluorimetric assays, the changes in LAP activity with increasing substrate concentration obeyed the Michaelis-Menten equation (Fig 2). The V_max_ value was 141 nmol AMC μg^-1^ enzyme h^-1^ for the purified LAP and ranged from 266 to 2660 nmol AMC g^-1^ soil h^-1^ for the soil LAP. The purified and soil LAP showed K_m_ values ranging from 37.7 to 58.5 μM. The variation in K_m_ values among the three soils may reflect differences in soil organic matter content, clay mineralogy, or pH (Table 1), which can influence enzyme-substrate binding affinity, as observed in other soil-substrate interaction studies [11,22]. The colorimetric assay using LNA yielded comparable V_max_ values (230 nmol β-naphthylamine μg^-1^ enzyme h^-1^ for the purified enzyme and 452–2190 nmol β-naphthylamine g^-1^ soil h^-1^ for the three soils) but much higher K_m_ values (191–1190 μM) compared with the fluorimetric assay (Figs 2 and S3).

**Fig 3:**
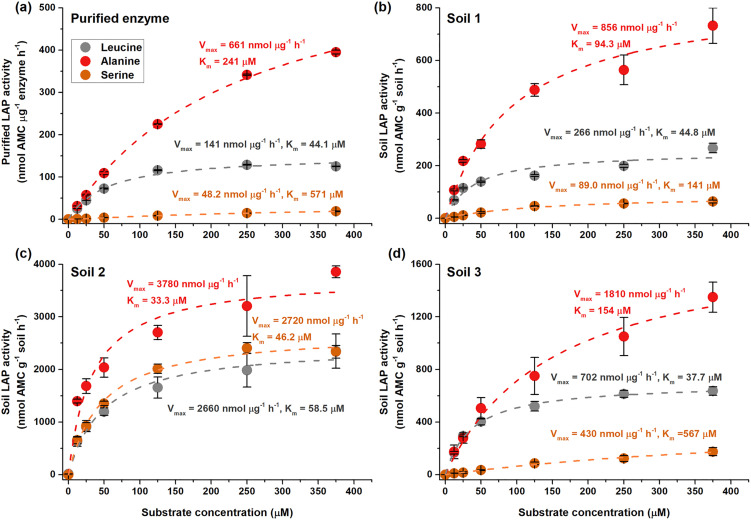
Specificity of LAP toward substrates with different amino acid moieties. Data are presented as mean ± standard error. The relationship between substrate concentration and enzyme activity was fitted by the Michaelis-Menten equation. The substrates used were leucine-AMC (grey dots), alanine-AMC (red dots) and serine-AMC (orange dots). Note that the unit and number range in the y-axis were not the same for the four panels. V_max_: maximum enzyme activity, K_m_: half saturation constant, the substrate concentration at which the enzyme activity equals V_max_/2, LAP: leucine aminopeptidase, AMC: 7-amido-4-methylcoumarin.

In the fluorimetric assays, the effects of the amino acid moiety of the substrate on the K_m_ and V_max_ values was similar for the purified LAP and the LAP in the Soils 1 and 3 (Fig 3a, 3b and 3d). The V_max_ values increased in the order of serine < leucine < alanine, while the K_m_ values increased in the order of leucine < alanine < serine. Soil 2 also showed the highest V_max_ values for alanine, but showed similar V_max_ values for leucine and serine (Fig 3c). Soil 2 showed similar K_m_ values for the three amino acid moieties (33.3–58.5 μM).

### 3.3. Temperature dependence

**Fig 4:**
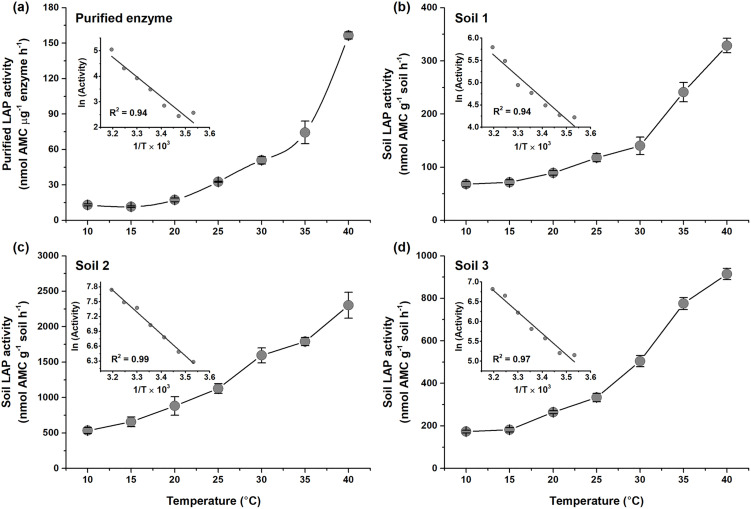
Effect of incubation temperature on LAP activity. Data are presented as mean ± standard error. The insets show the linear fitting of the change in LAP activity with temperature (Arrhenius equation, logarithmic form). Note that the unit and number range in the y-axis were not the same for the four panels. LAP: leucine aminopeptidase, AMC: 7-amido-4-methylcoumarin, T: absolute temperature (K).

In the fluorimetric assay, the changes in activities of the purified and soil LAP obeyed the Arrhenius equation in the temperature range of 10–40 °C (R^2^ = 0.94–9.99; Fig 4). The activity increased by 0.63–1.45 folds for each 10 °C increase in temperature (S1 Table). The activation energy was 64.07 kJ mol^-1^ for the reaction catalyzed by purified LAP and ranged from 36.73 to 44.79 kJ mol^-1^ for the reaction catalyzed by soil LAP (S1 Table). In the colorimetric assay using LNA as the substrate, the activation energy was 55.99 kJ mol^-1^ for the purified enzyme and ranged from 29.67 to 79.43 kJ mol^-1^ for the soil enzyme (S1 Table, S4 Fig).

### 3.4. Effects of incubation time and soil amount

**Fig 5:**
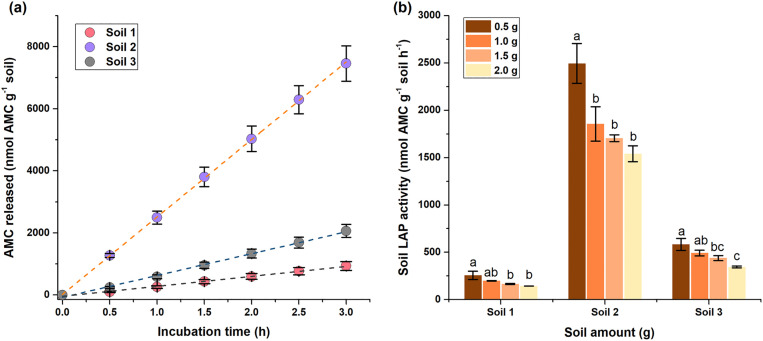
Effect of incubation time on the release of AMC during LAP assay (a) and effect of soil amount used in homogenate preparation on LAP activity (b). Data are presented as mean ± standard error. In panel **(a)**, the straight lines are the linear fitting of the accumulation of the AMC with incubation time. For each soil in panel (b), the different lowercase letters indicate significant differences among different soil amounts used in homogenate preparation. LAP: leucine aminopeptidase, AMC: 7-amido-4-methylcoumarin.

In the fluorimetric assay, the amount of released AMC increased linearly with incubation time for soil LAP (Fig 5a). When the amount of soil used to prepare the homogenate increased from 0.5 to 2.0 g per 120 mL water, the LAP activity significantly decreased by 44, 38 and 41% for Soil 1, Soil 2 and Soil 3, respectively (Fig 5b).

### 3.5. Effects of ions

The purified and soil LAP activities were inhibited by Cd^2+^ and Ag^+^ ions, and the inhibition effects increased from 10 to 1000 μM (S5 Fig). At 1000 μM, the inhibition rates were 92% (Cd^2+^) and 85% (Ag^+^) for the purified enzyme, and were 65–96% (Cd^2+^) and 56–90% (Ag^+^) for the three soils. Compared with the no-Co^2+^ treatment, the activity of the purified LAP increased by 23 and 22% at 50 and 100 μM of Co^2+^, respectively, while it decreased by 41 and 70% at 500 and 1000 μM, respectively. The LAP activity of Soil 1 was not affected by the Co^2+^ at concentrations up to 800 μM and it decreased by 44% at 1000 μM. For Soils 2 and 3, the LAP activities increased up to 49% after the addition of Co^2+^. The purified LAP was activated by the addition of Mn^2+^ (24 and 21% increase in activity at 100 and 250 μM), while the soil LAP was not affected (Soil 2) or inhibited (up to 41 and 18% decrease in activities for Soils 1 and 3, respectively) by Mn^2+^. The effects of Mg^2+^ and B_4_O_7_^2-^ on LAP activities were generally nonsignificant.

## 4. Discussion

### 4.1. Factors affecting the fluorimetric assay of soil LAP

Using a purified enzyme and three soils with contrasting properties, we demonstrated that LAP activity is highly sensitive to buffer pH, substrate concentration, and temperature. First, the optimal pH ranged from 7 to 9 (Table 1, Fig 1), which is consistent with a previous study involving five different soils [18] and aligns with earlier findings that most proteases have pH optima around 8 ± 1 [2]. The alkaline pH optimum of LAP implies that assays conducted at lower pH values (e.g., pH 5–6) may substantially underestimate activity. This is particularly relevant for agricultural soils where N fertilizer application can cause acidification [24]. In such acidified soils, researchers should be aware that measured LAP activity under standard buffer conditions may not reflect the actual potential activity of the enzyme, and pH-activity relationships should be characterized for specific soil types. Second, the reaction exhibited zero-order kinetics at a substrate concentration of 250 μM (approximately five times the K_m_ values) across the three soils, despite up to a 10-fold variation in their V_max_ values (Fig 2). Third, LAP activity increased as the temperature rose from 10 to 40 °C, corroborating reports from Luvisols sampled in central Germany (Fig 4; [19]), which suggests that the optimal temperature for LAP may be 40 °C or higher.

Despite the significant influence of these factors, widely cited studies on fluorimetric methods exhibit substantial variability in their assay conditions for LAP activity [8–10,12], potentially complicating cross-study comparisons and meta-analysis of measured soil LAP activities [11]. Furthermore, we observed that many studies conduct LAP activity measurements under suboptimal conditions. For instance, a buffer pH of 5 is frequently employed for the fluorimetric assay of soil LAP (e.g., [12], and many studies citing this work), yet LAP activity at this pH was an order of magnitude lower than at the optimal pH (Fig 1). Additionally, the use of unsaturated substrate concentrations and relatively low temperatures (e.g., 40 μM and 20 °C, [12]) may further diminish the assay sensitivity (Figs 2 and 4) and increase the risk of type II errors when evaluating treatment effects on soil LAP activity.

The effects of other factors were relatively weak but should also be considered in the fluorimetric assay of soil LAP. First, the linear increase in the amount of product (AMC) with incubation time (Fig 5a) indicates that the settling of soil particles during incubation did not hinder the contact between the enzyme and the substrate, and that microbial assimilation of AMC likely did not occur within the 3-hour incubation period [9,13]. Thus, the choice of incubation time represents a trade-off between high sensitivity (long incubation time) and practicality (short incubation time). Second, the measured LAP activity decreased with the amount of soil used to prepare the soil homogenate (Fig 5b). This may be due to insufficient homogenization of the soil-water mixture as the amount of soil increased, resulting in some particles settling at the bottom of the beaker and not being sampled by the multichannel pipette for the enzyme assay [25]. An additional, non-exclusive reason could be that a higher soil amount, which provides more reactive surfaces, might adsorb enzymes and/or the substrate, thereby reducing the measured LAP activity [21]. The adsorption of fluorogenic substrates and their fluorescent products onto soil particles may be molecular‑weight‑dependent, as observed for other organic compounds in environmental matrices [26]. Therefore, the choice of soil amount should balance the desired sensitivity with the representativeness of the soil sample for the enzyme assay [21]. Nonetheless, our results suggest that a consistent amount of soil should be used within one study. Third, we found no evidence that LAP activity can be substantially stimulated by the addition of ions (S5 Fig), indicating that cofactors may not be necessary for the fluorimetric assay of LAP.

### 4.2. Relationship between LAP and arylamidase

Our comparisons between the enzyme activities measured using leucine-AMC (LAP) and LNA (arylamidase) as substrates indicate that the two substrates may be targets of the same group of enzymes. This is supported by observations that both the purified enzyme and the soil enzymes exhibited similar pH optima (Figs 1 and S2), V_max_ values (Figs 2 and S3), and temperature dependence (Figs 4 and S4) when tested with the two substrates. Additionally, both purified LAP and soil LAP demonstrated higher activity for alanine-AMC compared to leucine-AMC and serine-AMC (Fig 3), a finding that has also been reported for soil arylamidase using β-naphthylamide substrates with various amino acid moieties [15]. Furthermore, the effects of different ions on enzyme activities were similar for both substrates, with strong inhibition observed from Cd²⁺ and Ag⁺ (S5 Fig; [14]).

However, we observed a significantly higher enzyme affinity (indicated by a lower K_m_ value) for leucine-AMC compared to LNA (Figs 2 and S3). Previous studies have reported affinities for the fluorogenic 4-methylumbelliferyl substrate that were up to two orders of magnitude higher than those for the chromogenic *p*-nitrophenyl substrate in soil enzyme assays [10]. The discrepancy in K_m_ values may be attributed to the differing adsorption behaviors of the fluorogenic and chromogenic substrates on soil particles because of their variations in properties including molecular weight [10]. To further test this hypothesis, future studies could integrate molecular-weight-resolved fractionation of dissolved substrates with controlled mineral surface adsorption experiments (e.g., adsorption isotherm measurements), followed by quantitative analysis of phase redistribution using Fourier transform infrared spectroscopy (FTIR) and Fourier transform ion cyclotron resonance mass spectrometry (FT-ICR MS), to elucidate size-dependent adsorption behavior of fluorogenic substrates and their fluorescent products onto soil particles [26,27]. Nevertheless, the K_m_ value for LNA was still more than four times greater than that for leucine-AMC in the case of the purified enzyme (Figs 2 and S3), suggesting that the aromatic group attached to the leucine residue may also influence the affinity between the enzyme and the substrate. Despite the factors contributing to the differing K_m_ values, the V_max_ values remained similar for both substrates (Figs 2 and S3). Since the measured activity approaches the V_max_ value under saturating substrate concentrations, we could obtain comparable enzyme activities using both substrates under similar assay conditions. This finding may facilitate the comparison of enzyme activities measured using fluorimetric and colorimetric methods, but it requires validation with a broader range of soils exhibiting different properties.

## 5. Conclusions

Based on the results of this study, the measured LAP activity in the fluorimetric assay was significantly influenced by buffer pH, substrate concentration, and temperature. Meanwhile, the activity of LAP was not substantially stimulated by the addition of metal ions, suggesting that a metal cofactor is not needed for the fluorimetric assay. We recommend using a substrate concentration of 250 μM or higher to ensure zero-order reaction kinetics. Additionally, we caution that researchers measuring soil LAP activity under field-relevant pH and temperature conditions may encounter low assay sensitivity and high risk of type II error, especially when the soil is acidic and the temperature is low. Furthermore, it is essential to use the same soil amount within a single study to effectively assess the treatment effects on soil LAP activity. While our findings require confirmation with a broader range of soils exhibiting different properties, they will contribute to the standardization of fluorimetric assay conditions for LAP, which is crucial for the meta-analysis of enzyme activities across various studies. Future developments in soil enzyme assays could explore ratiometric fluorescent probe strategies, which have shown potential for robust analyte detection in complex biological systems, such as living cells and in vivo models [28]. Although these approaches have not yet been directly validated for soil extracellular enzyme assays, their internal self-calibration capability may offer a promising route to mitigate signal variability caused by matrix effects, including particle scattering, fluorescence quenching, and heterogeneous soil backgrounds. Moreover, the LAP activity measured using leucine-AMC and the arylamidase activity measured colorimetrically using LNA may correspond to the same group of soil enzymes, which potentially facilitate comparisons of enzyme activities assessed through different methods.

## Supporting information

S1 Fig Response of AMC (7-amido-4-methylcoumarin) fluorescence to changes in incubation temperature. The AMC solution (0–50 μM) was incubated in 25 mM, pH 8 tris(hydroxymethyl)methyl aminomethane (THAM) buffer at 10–60 °C for 1 h, and the fluorescence was then measured within 1 min at room temperature. Four replicate wells were used for each AMC concentration at each temperature. The straight lines are the linear fitting of the florescence value with the AMC concentration. Note the significantly decreased fluorescence values at temperatures higher than 40 °C.
(DOCX)

S2 Fig Effect of buffer pH on arylamidase activity using L-leucine β-naphthylamide as substrate. Data are presented as mean ± standard error. Different lowercase letters indicate significant differences among different buffer pH values.
(DOCX)

S3 Fig Effect of substrate concentration on arylamidase activity using L-leucine β-naphthylamide as substrate. Data are presented as mean ± standard error. The relationship between substrate concentration and enzyme activity was fitted by the Michaelis-Menten equation. V_max_: maximum enzyme activity, K_m_: half saturation constant, the substrate concentration at which the enzyme activity equals V_max_/2.
(DOCX)

S4 Fig Effect of incubation temperature on arylamidase activity using L-leucine β-naphthylamide as substrate. Data are presented as mean ± standard error. The insets show the linear fitting of the change in LAP activity with temperature (Arrhenius equation, logarithmic form). T: absolute temperature (K).
(DOCX)

S5 Fig Response of LAP activity to ions of different concentrations. Data are presented as the ratio between the enzyme activity measured at an ion concentration with that measured at 0 concentration (mean ± standard error). The asterisks indicate significant difference between the enzyme activity measured at an ion concentration with that measured at 0 concentration. The ions were added as solutions of Co^2+^ (Co), Mn^2+^ (Mn), Mg^2+^ (Mg), Cd^2+^ (Cd), Ag^+^ (Ag) and B_4_O_7_^2-^ (B).
(DOCX)

S1 Table Activation energy (E_a_, kJ mol^-1^) and temperature coefficient (Q_10_) of the reactions using Leucine-AMC (fluorimetric assay) and LNA (colorimetric assay) as substrates. (DOCX)

S2 Table Raw data underlying the findings described in the manuscript. (XLSX)
