# Supplementary material for: Evaluation of fluorimetric assay conditions for measuring leucine aminopeptidase activity in soils
Source: PLoS One. 2026 Jul 7;21(7):e0352890. doi: 10.1371/journal.pone.0352890 (PMC13340760; doi:10.1371/journal.pone.0352890)
Supplement: S1 Fig — The AMC solution (0–50 μM) was incubated in 25 mM, pH 8 tris(hydroxymethyl)methyl aminomethane (THAM) buffer at 10–60 °C for 1 h, and the fluorescence was then measured within 1 min at room temperature. Four replicate wells were used for each AMC concentration at each temperature. The straight lines are the linear fitting of the florescence value with the AMC concentration. Note the significantly decreased fluorescence values at temperatures higher than 40 °C. (DOCX) [file pone.0352890.s001.docx]

**Fig. S1** Response of AMC (7-amido-4-methylcoumarin) fluorescence to changes in incubation temperature. The AMC solution (0-50 μM) was incubated in 25 mM, pH 8 tris(hydroxymethyl)methyl aminomethane (THAM) buffer at 10–60 °C for 1 h, and the fluorescence was then measured within 1 min at room temperature. Four replicate wells were used for each AMC concentration at each temperature. The straight lines are the linear fitting of the florescence value with the AMC concentration. Note the significantly decreased fluorescence values at temperatures higher than 40 °C.
