# Supplementary material for: Evaluation of fluorimetric assay conditions for measuring leucine aminopeptidase activity in soils
Source: PLoS One. 2026 Jul 7;21(7):e0352890. doi: 10.1371/journal.pone.0352890 (PMC13340760; doi:10.1371/journal.pone.0352890)
Supplement: S2 Fig — Data are presented as mean ± standard error. Different lowercase letters indicate significant differences among different buffer pH values. (DOCX) [file pone.0352890.s002.docx]

**Fig. S2** Effect of buffer pH on arylamidase activity using L-leucine β-naphthylamide as substrate. Data are presented as mean ± standard error. Different lowercase letters indicate significant differences among different buffer pH values.
