# Supplementary material for: Evaluation of fluorimetric assay conditions for measuring leucine aminopeptidase activity in soils
Source: PLoS One. 2026 Jul 7;21(7):e0352890. doi: 10.1371/journal.pone.0352890 (PMC13340760; doi:10.1371/journal.pone.0352890)
Supplement: S3 Fig — Data are presented as mean ± standard error. The relationship between substrate concentration and enzyme activity was fitted by the Michaelis-Menten equation. Vmax: maximum enzyme activity, Km: half saturation constant, the substrate concentration at which the enzyme activity equals Vmax/2. (DOCX) [file pone.0352890.s003.docx]

**Fig. S3** Effect of substrate concentration on arylamidase activity using L-leucine β-naphthylamide as substrate. Data are presented as mean ± standard error. The relationship between substrate concentration and enzyme activity was fitted by the Michaelis-Menten equation. V_max_: maximum enzyme activity, K_m_: half saturation constant, the substrate concentration at which the enzyme activity equals V_max_/2.
