# Supplementary material for: Evaluation of fluorimetric assay conditions for measuring leucine aminopeptidase activity in soils
Source: PLoS One. 2026 Jul 7;21(7):e0352890. doi: 10.1371/journal.pone.0352890 (PMC13340760; doi:10.1371/journal.pone.0352890)
Supplement: S4 Fig — Data are presented as mean ± standard error. The insets show the linear fitting of the change in LAP activity with temperature (Arrhenius equation, logarithmic form). T: absolute temperature (K). (DOCX) [file pone.0352890.s004.docx]

**Fig. S4** Effect of incubation temperature on arylamidase activity using L-leucine β-naphthylamide as substrate. Data are presented as mean ± standard error. The insets show the linear fitting of the change in LAP activity with temperature (Arrhenius equation, logarithmic form). T: absolute temperature (K).
