# Supplementary material for: Evaluation of fluorimetric assay conditions for measuring leucine aminopeptidase activity in soils
Source: PLoS One. 2026 Jul 7;21(7):e0352890. doi: 10.1371/journal.pone.0352890 (PMC13340760; doi:10.1371/journal.pone.0352890)
Supplement: S5 Fig — Data are presented as the ratio between the enzyme activity measured at an ion concentration with that measured at 0 concentration (mean ± standard error). The asterisks indicate significant difference between the enzyme activity measured at an ion concentration with that measured at 0 concentration. The ions were added as solutions of Co2+ (Co), Mn2+ (Mn), Mg2+ (Mg), Cd2+ (Cd), Ag+ (Ag) and B4O72- (B). (DOCX) [file pone.0352890.s005.docx]

**Fig. S5** Response of LAP activity to ions of different concentrations. Data are presented as the ratio between the enzyme activity measured at an ion concentration with that measured at 0 concentration (mean ± standard error). The asterisks indicate significant difference between the enzyme activity measured at an ion concentration with that measured at 0 concentration. The ions were added as solutions of Co^2+^ (Co), Mn^2+^ (Mn), Mg^2+^ (Mg), Cd^2+^ (Cd), Ag^+^ (Ag) and B_4_O_7_^2-^ (B).

**Fig. S5-1** Response of purified LAP activity to ions of different concentrations. Data are presented as the ratio between the enzyme activity measured at an ion concentration with that measured at 0 concentration (mean ± standard error). The asterisks indicate significant difference between the enzyme activity measured at an ion concentration with that measured at 0 concentration. The ions were added as solutions of Co^2+^ (Co), Mn^2+^ (Mn), Mg^2+^ (Mg), Cd^2+^ (Cd), Ag^+^ (Ag) and B_4_O_7_^2-^ (B).

**Fig. S5-2** Response of LAP activity in Soil 1 to ions of different concentrations. Data are presented as the ratio between the enzyme activity measured at an ion concentration with that measured at 0 concentration (mean ± standard error). The asterisks indicate significant difference between the enzyme activity measured at an ion concentration with that measured at 0 concentration. The ions were added as solutions of Co^2+^ (Co), Mn^2+^ (Mn), Mg^2+^ (Mg), Cd^2+^ (Cd), Ag^+^ (Ag) and B_4_O_7_^2-^ (B).

**Fig. S5-3** Response of LAP activity in Soil 2 to ions of different concentrations. Data are presented as the ratio between the enzyme activity measured at an ion concentration with that measured at 0 concentration (mean ± standard error). The asterisks indicate significant difference between the enzyme activity measured at an ion concentration with that measured at 0 concentration. The ions were added as solutions of Co^2+^ (Co), Mn^2+^ (Mn), Mg^2+^ (Mg), Cd^2+^ (Cd), Ag^+^ (Ag) and B_4_O_7_^2-^ (B).

**Fig. S5-4** Response of LAP activity in Soil 3 to ions of different concentrations. Data are presented as the ratio between the enzyme activity measured at an ion concentration with that measured at 0 concentration (mean ± standard error). The asterisks indicate significant difference between the enzyme activity measured at an ion concentration with that measured at 0 concentration. The ions were added as solutions of Co^2+^ (Co), Mn^2+^ (Mn), Mg^2+^ (Mg), Cd^2+^ (Cd), Ag^+^ (Ag) and B_4_O_7_^2-^ (B).
