# Supplementary material for: Evaluation of fluorimetric assay conditions for measuring leucine aminopeptidase activity in soils
Source: PLoS One. 2026 Jul 7;21(7):e0352890. doi: 10.1371/journal.pone.0352890 (PMC13340760; doi:10.1371/journal.pone.0352890)
Supplement: S1 Table — (DOCX) [file pone.0352890.s006.docx]

**Table S1** Activation energy (E_a_, kJ mol^-1^) and temperature coefficient (Q_10_)^a^ of the reactions using Leucine-AMC (fluorimetric assay) and LNA (colorimetric assay) as substrates

|  | Fluorimetric assay | | | | | | Colorimetric assay | | | | | |
| --- | --- | --- | --- | --- | --- | --- | --- | --- | --- | --- | --- | --- |
|  | E_a_ | R^2^ | Q_10_ of the temperature indicated | | | | E_a_ | R^2^ | Q_10_ of the temperature indicated | | | |
|  |  |  | 20 °C | 30 °C | 40 °C | mean |  |  | 20 °C | 30 °C | 40 °C | mean |
| Purified enzyme | 64.07 | 0.94 | 1.32 | 2.94 | 3.08 | 2.45 | 55.99 | 0.99 | 1.99 | 2.48 | 1.80 | 2.09 |
| Soil 1 | 39.77 | 0.94 | 1.31 | 1.57 | 2.34 | 1.74 | 63.61 | 0.97 | 3.22 | 2.19 | 1.71 | 2.37 |
| Soil 2 | 36.73 | 0.99 | 1.65 | 1.81 | 1.44 | 1.63 | 29.67 | 0.96 | 1.18 | 1.90 | 1.39 | 1.49 |
| Soil 3 | 44.79 | 0.97 | 1.53 | 1.91 | 1.81 | 1.75 | 79.43 | 0.94 | 4.91 | 2.11 | 2.20 | 3.07 |

^a^Q_10_ is calculated following Equation 1.

$Q_{10}=\frac{Enzyme activity at given temperature}{Enzyme activity at given temperature-10 ℃}$ (1)
