# Supplementary material for: Evaluation of fluorimetric assay conditions for measuring leucine aminopeptidase activity in soils
Source: PLoS One. 2026 Jul 7;21(7):e0352890. doi: 10.1371/journal.pone.0352890 (PMC13340760; doi:10.1371/journal.pone.0352890)
Supplement: Attachment — Submitted filename: Response to Reviewers.docx [file pone.0352890.s008.docx]

**1. Responses to comments from the Academic Editor**

**Comment 1**: Thank you for submitting your manuscript to PLOS ONE. After careful consideration, we feel that it has merit but does not fully meet PLOS ONE’s publication criteria as it currently stands. Therefore, we invite you to submit a revised version of the manuscript that addresses the points raised during the review process.

**Response**: Thank you very much for your valuable suggestions on our manuscript. We sincerely appreciate that you gave us the opportunity to revise it. In response to the constructive comments from you and the reviewer, we have made comprehensive modifications to the paper and believe that we have adequately addressed all concerns raised. Below, we provide point-by-point responses to each comment.

**Comment 2**: To the manuscript, provide an experimental method to support the evidence of molecular‑weight‑dependent control of the adsorption of fluorogenic substrates and their fluorescent products onto soil particles.

**Response**: We greatly appreciate your insightful suggestions. Following your comments, we have added prospective experimental methods for future investigations in the revised manuscript, which can help verify how molecular weight regulates the adsorption of fluorogenic substrates and their fluorescent products on soil particles. As quantitative measurement of the sorption properties of substrates and products is beyond the scope of this work, we have presented these methodologies for future reference in the Discussion section (*L. 309-339 in the revised manuscript*). We will gladly revise the manuscript further based on your feedback.

**2. Responses to journal requirements**

**Comment 1**: Please ensure that your manuscript meets PLOS ONE’s style requirements, including those for file naming. The PLOS ONE style templates can be found at https://journals.plos.org/plosone/s/file?id=wjVg/PLOSOne_formatting_sample_main_body.pdf and https://journals.plos.org/plosone/s/file?id=ba62/PLOSOne_formatting_sample_title_authors_affiliations.pdf

**Response**: We have carefully reviewed PLOS ONE’s style requirements and updated the manuscript accordingly, including file naming, formatting, and reference style.

**Comment 2**: In your Methods section, please provide additional information regarding the permits you obtained for the work. Please ensure you have included the full name of the authority that approved the field site access and, if no permits were required, a brief statement explaining why.

**Response**: We have added a statement in the Methods section confirming that soil samples were collected from research stations of the Chinese Ecosystem Research Network (CERN) and the Mulun National Nature Reserve with the appropriate permissions (*L. 80-83 in the revised manuscript*).

**Comment 3**: We note that the grant information you provided in the ‘Funding Information’ and ‘Financial Disclosure’ sections do not match. When you resubmit, please ensure that you provide the correct grant numbers for the awards you received for your study in the ‘Funding Information’ section.

**Response**: We have carefully checked and corrected the grant information to ensure consistency between the ‘Funding Information’ and ‘Financial Disclosure’ sections.

**Comment 4**: In the online submission form, you indicated that [Data are available from the corresponding author upon reasonable request.]. All PLOS journals now require all data underlying the findings described in their manuscript to be freely available to other researchers, either 1. In a public repository, 2. Within the manuscript itself, or 3. Uploaded as supplementary information. This policy applies to all data except where public deposition would breach compliance with the protocol approved by your research ethics board. If your data cannot be made publicly available for ethical or legal reasons (e.g., public availability would compromise patient privacy), please explain your reasons on resubmission and your exemption request will be escalated for approval.

**Response**: We have prepared the raw data underlying all figures and tables for upload as Supporting Information files. The data availability statement in the manuscript has been updated accordingly to reflect how the data can be accessed (*Table S2 Raw data underlying the findings described in the manuscript*).

**Comment 5**: Please include captions for your Supporting Information files at the end of your manuscript, and update any in-text citations to match accordingly. Please see our Supporting Information guidelines for more information: http://journals.plos.org/plosone/s/supporting-information.

**Response**: We have added captions for all Supporting Information files at the end of the manuscript and updated in-text citations to match (*L. 370-397 in the revised manuscript*).

**Comment 6**: If the reviewer comments include a recommendation to cite specific previously published works, please review and evaluate these publications to determine whether they are relevant and should be cited. There is no requirement to cite these works unless the editor has indicated otherwise.

**Response**: We have carefully reviewed all recommended citations. For those that are relevant to our study, we have incorporated them into the revised manuscript. For several suggested references that appear to address topics unrelated to soil enzyme assay methodology, we have provided detailed reasoning in our individual responses to the specific reviewer comments below and cited proper references. We are happy to make further adjustments based on your guidance.

**3. Responses to comments from the reviewer**

**Comment 1**: The authors mention that LAP is used as an indicator of microbial investment in nitrogen (N) acquisition. However, they do not cite recent work on soil ecological stoichiometry that uses LAP alongside other enzymes (e.g., β-1,4-N-acetylglucosaminidase, alkaline phosphatase) to assess microbial nutrient limitation. Adding such context would increase the paper’s relevance to soil ecology and biogeochemistry. At Section 1 (Introduction), after the mention of LAP as an indicator of microbial N acquisition, insert: “Standardised LAP assays are particularly important for soil ecological stoichiometry studies, where LAP activity is often compared with C‑ and P‑acquiring enzymes to infer microbial nutrient limitation across ecosystems (Liu, et al. 2023).” [Liu, J., et al. (2023). Soil ecological stoichiometry synchronously regulates stream nitrogen and phosphorus concentrations and ratios. CATENA, 231, 107357. DOI: https://doi.org/10.1016/j.catena.2023.107357]

**Response**: We thank the reviewer for this valuable suggestion. We have added the recommended text about soil ecological stoichiometry to the Introduction section, after the sentence mentioning LAP as an indicator of microbial N acquisition (*Line 43-45 in the revised manuscript*).

**Comment 2**: The authors use a purified LAP from porcine kidney. However, they do not discuss whether soil LAP from microbial sources might have different kinetic properties or optimal conditions. A brief note on the potential limitations of using animal‑derived purified enzymes as a proxy for soil microbial enzymes would strengthen the Discussion. At Section 4 (Discussion), after the comparison of purified and soil LAP behaviours, Add: “While the purified porcine LAP provided a useful reference, soil LAP originates from diverse microbial communities and may exhibit different kinetic properties; careful validation of assay conditions for soil matrices remains essential, as in other fluorogenic detection systems (Gong, et. al. 2025).” [Gong, Z., et al. (2025). A one-pot isothermal Fluorogenic Mango II arrays-based assay for label-free detection of miRNA. Talanta, 281, 126920. DOI: https://doi.org/10.1016/j.talanta.2024.126920]

**Response**: We thank the reviewer for this important point. We have added a brief note discussing the limitations of using animal-derived purified LAP as a proxy for soil microbial enzymes (*Line 120-124 in the revised manuscript*). As this is a caution regarding the method used, we added the note to the Materials and Methods section instead of the Discussion section. The added text acknowledges that microbial LAP enzymes can differ from animal-derived enzymes in optimal pH, temperature sensitivity, and cofactor requirements depending on the taxonomic origin and ecological niche of the producing microorganisms, and that future studies using purified microbial LAP enzymes or molecular approaches would help bridge this gap. However, the suggested reference (Gong et al., 2025, Talanta, 281, 126920: “A one-pot isothermal Fluorogenic Mango II arrays-based assay for label-free detection of miRNA”) describes an in vitro miRNA detection method using fluorogenic RNA arrays. It does not address soil enzymes, aminopeptidases, or enzyme kinetics. We have therefore not cited this reference and have instead cited German et al. (2011) (Soil Biology and Biochemistry, 43 (2011) 1387-1397: “Optimization of hydrolytic and oxidative enzyme methods for ecosystem studies”).

**Comment 3**: Abstract, line 31: “they may represents the same group” – change “represents” to “represent” (*Line 31 in the revised manuscript*).

**Response**: Thank you for your suggestion. We have revised the text accordingly.

**Comment 4**: Page 8, line 58: “Given that leucine AMC is similar to LNA” – change “Given that” to “Because”.

**Response**: Thank you for your careful reading. We have revised the text accordingly (*Line 60 in the revised manuscript*).

**Comment 5**: Page 12, line 99: “tris(hydroxymethyl)methyl aminomethane (THAM) buffer” – define the abbreviation THAM at first use (it is defined, but ensure consistency).

**Response**: Thank you for your suggestion. We have defined the abbreviation THAM at first use (*Line 104 in the revised manuscript*).

**Comment 6**: The authors observe that LAP activity is inhibited by increasing soil amount, possibly due to adsorption of enzymes or substrates onto soil particles. This adsorption behaviour may be molecular‑weight‑dependent, a concept explored in recent environmental chemistry studies. Section 4 (Discussion), after the discussion of soil amount effects, consider adding: “The adsorption of fluorogenic substrates and their fluorescent products onto soil particles may be molecular‑weight‑dependent, as observed for other organic compounds in environmental matrices (Xu, et al. 2026).” [Xu, C., et al. (2026). Molecular-weight-dependent mixing behavior of organic phosphorus and carbon in a subtropic river-estuary-bay continuum. Water Research, 296, 125650. DOI: https://doi.org/10.1016/j.watres.2026.125650]

**Response**: We thank the reviewer for this insightful suggestion. We have added a discussion of molecular-weight-dependent adsorption in Section 4.1, after the discussion about soil amount effects. The added text discusses how larger molecules with higher molecular weights may experience greater adsorption to soil mineral and organic surfaces, which could complicate the comparison of enzyme activities measured with different substrates. We have cited Xu et al. (2026) as suggested (*Line 309-311 in the revised manuscript*).

**Comment 7**: The authors report Km values for the three soils that vary considerably (37.7–58.5 μM for leucine-AMC). They do not discuss whether these differences are related to soil properties (e.g., organic matter content, clay content, pH). Adding a brief analysis or hypothesis would strengthen the interpretation. At Section 3.2 (Kinetic parameters and substrate specificity), after the presentation of Km values, Add: “The variation in Km values among the three soils (37.7–58.5 μM) may reflect differences in soil organic matter content, clay mineralogy, or pH, which can influence enzyme–substrate binding affinity, as observed in other soil–substrate interaction studies (Wang, et al. 2024).” [Wang Fengchi, et al. (2024). Research on engineering characteristics of magnesium phosphate cement solidified copper contaminated soil. Journal of Shenyang Jianzhu University (Natural Science), 40(2), 313–321. DOI: 10.11717/j.issn:2095-1922.2024.02.14]

**Response**: We thank the reviewer for this constructive comment. We have added a discussion of the variation in Km values in Section 3.2, relating these differences to soil properties including organic matter content, clay mineralogy, and pH. The added text explains how soils with higher organic matter content may provide more adsorption sites that compete with the enzyme for substrate binding, potentially increasing the apparent Km, and how differences in clay content and mineralogy can affect the availability of both enzyme and substrate in the aqueous phase. However, the suggested reference (Wang Fengchi et al., 2024, Journal of Shenyang Jianzhu University (Natural Science), 40(2), 313-321: “Research on engineering characteristics of magnesium phosphate cement solidified copper contaminated soil”) is a civil/geotechnical engineering study and is unrelated to soil enzyme kinetics, enzyme-substrate interactions, or soil biochemistry. We have therefore not cited this reference and cited more relevant studies instead (Nannipieri P, Trasar-Cepeda C, Dick RP. Soil enzyme activity: a brief history and biochemistry as a basis for appropriate interpretations and meta-analysis. Biology and Fertility of Soils. 2018;54:11-9; Tabatabai M. Soil enzymes. In: Hart SC, Stark JM, Davidson EA, Firestone MK, editors. Methods of soil analysis Part 2 Microbiological and Biochemical Properties. 2. Madison, Wisconsin, USA: Soil Science Society of America; 1994. p. 775-833) (*Line 200-203 in the revised manuscript*).

**Comment 8**: Page 18, line 193: “The colorimetric assay using LNA showed comparable Vmax values” – change “showed” to “yielded”.

**Response**: Thank you for this suggestion. We have made the change in the revised manuscript (*Line 203 in the revised manuscript*).

**Comment 9**: Page 19, line 282: “the choice of incubation time represents a tradeoff” – change “tradeoff” to “trade‑off” (hyphenated).

**Response**: Thank you for this suggestion. We have made the change in the revised manuscript (*Line 302 in the revised manuscript*).

**Comment 10**: The authors use a standard fluorogenic substrate (leucine-AMC) that releases a fluorescent product (AMC). Recent advances in ratiometric fluorescent probes could offer alternatives for soil enzyme assays, particularly for reducing matrix interference. A brief mention of these emerging technologies would position the study within broader analytical chemistry developments. As a future direction, add: “Future developments in soil enzyme assays could explore ratiometric fluorescent probes, which have shown promise in reducing matrix interference in complex biological samples (Xu, et al. 2023).” [Xu, H., et al. (2023). A lysosome-targetable fluorescent probe for the ratiometric detection of formaldehyde in living cells and in vivo. Organic & Biomolecular Chemistry, 21(31), 6405–6409. DOI: 10.1039/D3OB00888F]

**Response**: We thank the reviewer for this forward-looking suggestion. We have added a sentence in Conclusions section about the potential of ratiometric fluorescent probes for reducing matrix interference in soil enzyme assays, citing Xu et al. (2023). We contextualized the citation by noting that although these approaches have not yet been directly validated for soil extracellular enzyme assays, their internal self-calibration capability may offer a promising route to mitigate signal variability caused by matrix effects, including particle scattering, fluorescence quenching, and heterogeneous soil backgrounds (*Line 360-366 in the revised manuscript*).

**Comment 11**: The authors find that LAP has an alkaline pH optimum (7–9). This has implications for measuring LAP activity in agricultural soils that are often acidified by nitrogen fertiliser application. A brief discussion of how soil acidification might affect measured LAP activity and its interpretation would add practical value. At Section 4 (Discussion), after the pH optimum results, insert: “The alkaline pH optimum of LAP implies that assays conducted at lower pH values (e.g., pH 5–6) may substantially underestimate activity, which is particularly relevant for agricultural soils where nitrogen fertiliser application can cause acidification (Wang et al. 2024).” [Wang, F., et al. (2024). Arsenic mobilization and nitrous oxide emission modulation by different nitrogen management strategies in a flooded ammonia-enriched paddy soil. Pedosphere, 34(6), 1051–1065. DOI: https://doi.org/10.1016/j.pedsph.2023.09.008

**Response**: We thank the reviewer for this practical and important suggestion. We have added a brief discussion in Section 4.1, after the discussion of pH optima, addressing the implications of the alkaline pH optimum for agricultural soils subject to N fertilizer-induced acidification. The added text notes that in acidified agricultural soils, LAP activity measured under standard buffer conditions may not reflect the actual potential activity of the enzyme, and that researchers should characterize pH-activity relationships for their specific soil types. We have cited Wang et al. (2024) as suggested (*Line 276-281 in the revised manuscript*).
